# Refining index to measure physical activity inequality: which group of the population is the most vulnerable?

**DOI:** 10.1186/s12939-022-01725-1

**Published:** 2022-08-31

**Authors:** Dyah Anantalia Widyastari, Aunyarat Khanawapee, Wanisara Charoenrom, Pairoj Saonuam, Piyawat Katewongsa

**Affiliations:** 1 Institute for Population and Social Research, Mahidol University, Salaya, Phutthamonthon, Nakhon Pathom, 73170 Thailand; 2 Thailand Physical Activity Knowledge Development Centre (TPAK), Institute for Population and Social Research, Mahidol University, Salaya, Phutthamonthon, Nakhon Pathom, 73170 Thailand; 3 Thai Health Promotion Foundation, Bangkok, Thailand

**Keywords:** PA inequality, Covid-19 epidemic, MVPA, Vulnerable population

## Abstract

**Background:**

The existing body of research mostly discusses inequality in physical activity (PA) based on the difference in the level of moderate-to-vigorous physical activity (MVPA). Evidence is lacking on the quantified inequality measures (e.g., how big the inequality is, and the distribution) in order to identify the most vulnerable groups of a population. This study measured PA inequality among Thai adults by using three parameters to construct an inequality index: (1) Proportion of the population with sufficient MVPA; (2) Cumulative minutes of MVPA; and (3) The Gini coefficient.

**Methods:**

This study employed three rounds of data from Thailand’s Surveillance on Physical Activity (SPA) 2019–2021. In each round, over 6,000 individuals age 18–64 years were selected as nationally-representative samples, and were included in the analysis. PA inequality was constructed by using three parameters, with a combination of the three as the final measure, to identify the sub-groups of the Thai adults who are most vulnerable: groups with the least MVPA, highest insufficiency, and highest inequality index (Gini).

**Results:**

Covid-19 containment measures have widened the gap in PA inequality, as shown by a declining proportion of the population meeting the recommended guidelines, from 74.3% in 2019 to 56.7% in 2020 and 65.5% in 2021. PA inequality existed in all sub-populations. However, by combining three parameters, the most vulnerable groups during the Covid-19 epidemic were identified as follows: (1) Those with no income; (2) The unemployed; (3) Those who have no access to PA facilities; (4) Older adults aged 60 + years; and (5) Those earning < 3,500 baht per month. Further, residents of Bangkok, young adults aged 18–24, individuals who attained primary level education or less, those who had no exposure to a PA awareness campaign and those who have a debilitating chronic disease also had elevated risk of PA insufficiency.

**Conclusion:**

A concerning level of PA inequality existed in all sub-populations. The use of combined indicators in measuring PA inequality should aid in determining the most vulnerable groups of the population with a refined procedure. This method can be applied in many settings since the baseline data used to measure inequality (i.e., percent sufficient and cumulative minutes of MVPA) are widely available.

## Introduction

Inequality in health has become a global research agenda as it reflects a country’s performance in achieving its developmental goals. Likewise, the discussion of inequality in physical activity (PA) has become of great interest, considering the high prevalence of inactivity across the globe [1]. There is substantial evidence that inequality – both in access and opportunity – is a predominant factor affecting one’s participation in PA [2–6]. With the emergence of Covid-19 in late 2019, and harsh government measures to contain the virus (e.g., lockdown, restriction of movement, closures of public and private facilities), PA inequality has widened [7–9]. The inequality is more profound, as the socio-economic turmoil from Covid-19 pandemic has disrupted the lives and livelihoods of individuals, families, and society worldwide [9–11]. Previous findings indicate that the socially and economically under-privileged population has lost most of their access to PA facilities, experienced a significant decline in their PA, and have increased sedentary behavior during strict Covid-19 containment measures [7, 8, 12]. To address this issue, WHO launched a ‘*Fair Play’* campaign that calls for collective action to address PA inequality and promoted equitable access and opportunities for everyone [13].

It should be noted however, that the evidence of PA inequality is mostly focused on the disparities in the level (prevalence of sufficiency or cumulative minutes) of MVPA classified by age, sex, socio-economic status (SES), and geographic region. Gender inequality in PA was frequently documented as lower prevalence of sufficient MVPA among females compared to males [1]. In terms of age group, adolescents and the oldest adults (60 + years) were mostly identified as the least active [14, 15]. Socio-economic inequality in PA also has been frequently reported. Individuals from lower SES (i.e., lower level of education) were more likely to engage in work-related PA and have less opportunity for recreational PA compared to their higher-SES counterparts [16–18]. Regional disparities are reflected by a higher level of inactivity among Latin American, Central Asian, and northern-hemisphere countries [1]. Previous studies in Thailand also reported that the PA level of Thais differed according to age, sex, geographical region, residential area (urban/rural), educational attainment, and occupation [19]. PA participation was also correlated with physical and social environment of the individual, including family, peers, and the availability of facilities and information [20, 21].

Because the existing studies mostly address PA inequality based on its differentials in the level, there is a lack of evidence on different dimensions of inequality. For example, how large is the inequality in PA, and how is PA distributed among sub-populations? This information is important in order to prioritize which groups of the population are the most vulnerable, i.e., with the least MVPA. To answer those questions, a more refined quantification of inequality is needed. Measures of inequality were firstly developed by economists to assess inequality in income distribution [22]. Other metrics have been developed as practical tools in measuring inequality, including the Coefficient of Variation [23, 24], the Concentration Index (CI) [11], Decile Ratios [25], the Generalized Entropy (GE) Index [26], and the Atkinson Index [27], among others. Of these, the Gini Coefficient is the most widely used index to quantify inequality in the public health sphere.

To date, there have been only a few studies measuring PA inequality by using a quantified inequality index. Those studies mostly focused on income inequality and how it affects PA participation [28], or how the environmental disparities within a sub-national entity (i.e., a state) affected a population’s PA [29–31]. PA disparities between countries were also compared [32], but most of those studies were conducted before the Covid-19 pandemic. Furthermore, most inequality measures relied on a single indicator, e.g., prevalence of sufficient MVPA (meeting the WHO guidelines) [5], cumulative minutes of MVPA [33] or steps/day [34]. Therefore, this study aimed to assess PA inequality among Thai adults by using three parameters to construct the inequality index: 1) Proportion of the population with sufficient MVPA; 2) Cumulative minutes of MVPA; and 3) The Gini coefficient. The first metric showed the proportion of individuals in a particular group who meet the WHO recommended guidelines on PA, and can be used directly to observe inequalities between-groups (i.e., males vs females). The second metric assessed the average minutes of MVPA that individuals collected per week, which also indicated their opportunities in PA participation. Similarly, this metric also can be used directly to observe between-group differences. As the third metric, the Gini coefficient showed the distribution of cumulative minutes of MVPA in a particular group of population that pointed inequalities within sub-population (i.e., within male group). The metrics used in this study should be considered as refined measures of inequality which employ a new method of assessment by calculating the level of PA of each parameter and of the combined value. The final determination of the most vulnerable population involved combining the three parameters. Various population characteristics over three rounds of surveys (2019–21) were also included to compare PA inequality within and between sub-populations, and before and during the sequential waves of Covid-19 epidemic. The findings of this study should be beneficial for the government and policy makers to gain understanding of the level of PA inequality, and to identify the groups of the population that require the most attention in order to design future PA policies and programs toward a more equitable society.

## Methods

### Data, population and sample

This study employed data from three rounds of Thailand’s Surveillance on Physical Activity (SPA) 2019–2021. The SPA itself is a nationally-representative survey that is conducted annually to collect information on PA of the Thai population age five years or older by using multi-stage stratified random sampling and considering place of residence, sex, and age group. However, to match the sample of SPA2020-2021, only persons age 18 + years who had access to the Internet were considered eligible. A total of 6,331 individuals from five geographic regions, 13 provinces, and 36 villages were selected from the SPA2019 sample.

During the Thai government’s containment measures (i.e., mobility restriction, curfew, closure of public facilities) to prevent the spread of Covid-19, SPA2020 and 2021 were designed as an online survey, involving nationally-representative samples of persons age 18 + years who had access to the Internet. The online population was estimated from the National Statistical Office data, classified by province. Samples were drawn randomly from Facebook pages, stratified by their location (i.e., district of residence). The Facebook users were invited to participate in the survey in a systematic random technique, by determining the starting point and then selecting every *i* ^th^ items on the sampling frame based on a certain interval [35]. Inclusion criteria include having an unambiguous gender in their profile, and aged 18–64. A total of 6,756 and 6,344 individuals were selected for the analysis from SPA2020 and SPA2021, respectively.

In all SPAs (face-to-face or online survey), PA was measured subjectively by using the Global Physical Activity Questionnaire (GPAQ) version 2.0 [36]. The GPAQ was converted into a Thai version, and subjected to a validity test in 2013 [12] by comparing the PA level between data from the questionnaire and an accelerometer. A significant correlation was detected from Pearson’s score and the Bland Altman coefficient [37], indicating that the GPAQ Thai version was a reliable instrument to measure PA of the Thai population. PA in this study is expressed by the following: (1) Minutes (denoting the average cumulative minutes of MVPA per week); and (2) Sufficient MVPA, with the cut-off points following WHO 2020 guidelines [38].

### Measurements and analysis

The inequality measures in this study were used to calculate the level of PA inequality, and determining the most vulnerable population from the three parameters. The first parameter is the proportion of population with “*sufficient*” MVPA. This study refers to the WHO recommendation on “*sufficien*t” level of PA for persons age 18–64 years as follows: 150–300 min of moderate-intensity aerobic PA, or at least 75–150 min of vigorous-intensity aerobic PA, or an equivalent combination of moderate- and vigorous-intensity activity throughout the week [38]. PA inequality based on sufficient MVPA was defined as the difference in the proportion of the population meeting the WHO guidelines, classified by various population characteristics. From this method, the PA inequality between groups can be determined. The most vulnerable population was defined as the group for which the gap between the highest and the lowest proportion of PA sufficiency was the highest.

The second parameter is inequality in the cumulative minutes of MVPA. This was defined as the difference in the mean cumulative weekly minutes of MVPA. This indicator was also classified by various population characteristics in order to compare inequality between sub-populations. The most vulnerable population was defined as the group with the largest gap between the highest and lowest cumulative minutes of MVPA.

The third measure of PA inequality in this study used the Gini coefficient. For the purposes of this study, the Gini Coefficient was calculated as a summary statistic of the Lorenz curve of cumulative percentage of minutes MVPA against the population distribution [39]. More specifically, it is the ratio between the equality line and the Lorenz curve (i.e., total area under the equality line) [34]. The potential value of the Gini Coefficient ranges from 0 to 1, with 0 denoting complete equality, and 1 denoting complete inequality. However, for this study, a value less than 0.3 was considered as a particularly equitable condition, 0.3–0.4 as a normal condition, while a value higher than 0.4 raises concern, and a value greater than 0.6 indicates a dangerous state of inequality [40]. The Gini coefficient itself can be used to observe within- and between-group inequality. In addition, to compare inequality between pre-Covid and during the Covid-19 epidemic, three rounds of survey data (SPA2019-2021) were used. The relative difference of the Gini coefficient (between base year to 2020 and 2021) is presented to show the effect of the Covid-19 epidemic on the population’s MVPA in two different time periods.

The fourth measure of PA inequality combined the above three parameters by firstly averaging the ranks of each sub-population. For the two parameters (proportion of sufficient MVPA and cumulative minutes), a sub-population was ranked from low to high. For the Gini coefficient, the sub-population was ranked from high to low. We then averaged the ranks to define the most vulnerable population sub-group by identifying the ten lowest.

Additionally, we graphed the Lorenz curve to show the distribution of overall PA within the population. The horizontal (x) axis displays the cumulative distribution of the population (persons age 18 + years), and the vertical (y) axis represents the distribution of population MVPA (in minutes). The cumulative minutes of MVPA was first ranked in order (from low to high) before being graphed as a continuous distribution. The Lorenz curve was drawn by plotting along the two axes: Cumulative percentage of the population and the cumulative percentage of minutes MVPA. Potential values on the x and y axes range from 0 to 1, but can also be expressed as a percentile (1 to 100%). The complete equality line is drawn as a diagonal, with a slope of 1 (45 degrees).

As correlates, we included sex, age group, region of residence, area of residence (urban/rural), occupation, education, income and whether the sample has a debilitating chronic disease. To assess the effectiveness of government’s health promotion messages, we asked the respondents if they ever heard any PA awareness campaign (i.e., Fit from Home, virtual running and cycling, safe park run, and benefits of PA) during the Covid-19 restriction measures, and whether they followed the recommendation. We also asked the respondents whether they have any access to PA facilities nearby their home. Microsoft Excel was used to calculate Gini coefficient, whereas Chi-square test, independent t-test and one way ANOVA (SPSS) were used to analyze the relative differences (between and within group) with a significant level of 0.05 or lower to determine the association.

## Results

The SPA samples comprise almost equal proportions between men and women. However, more than half were age 25–59 years old. While the proportion between urban and rural residents was almost equal in SPA2019 (54.1 vs 45.9%), more urban residents (66.1 and 66%) participated in the 2020 and 2021 study. SPA2019 has predominantly primary and secondary school graduates, and only 20% completed a higher level of education, whereas about half (50.9 and 48.1%) of SPA2020 and 2021 respondents attained a post-secondary degree (Table 1).

**Table 1:** Sample characteristics

Characteristics	SPA 2019 (*n* = 6,331)		SPA 2020 (*n* = 6,756)		SPA 2021 (*n* = 6,344)	
	n	%	n	%	n	%
***Sex***						
Male	3,052	48.2	3,422	50.7	3,053	48.1
Female	3,279	51.8	3,334	49.3	3,291	51.9
***Age group (years)***						
Yong adult (18–24)	620	9.8	1,383	20.5	978	15.4
Prime working age (25–59)	4,217	66.6	4,953	73.3	4,210	66.4
Older adults (60 +)	1,494	23.6	420	6.2	1,156	18.2
***Region***						
North	1,343	21.2	1,885	27.9	1,729	27.3
Central	1,468	23.2	2,060	30.5	2,030	32.0
Northeast	1,588	25.1	1,039	15.4	837	13.2
South	1,385	21.9	964	14.2	838	13.2
Bangkok	547	8.6	808	12.0	910	14.3
***Area of residence***						
Urban	3,427	54.1	4,464	66.1	4,187	66.0
Rural	2,904	45.9	2,292	33.9	2,157	34.0
***Occupation***						
Agriculture	1,032	16.3	447	6.6	577	9.1
Formal sector	1,130	17.9	2,633	39.0	2,303	36.3
Informal sector	2,642	41.7	2,802	41.5	2,204	34.7
Unemployed	1,526	24.1	763	11.3	1,117	17.6
No response	1	0.0	111	1.6	134	2.3
***Education***						
Primary or less	2,731	43.2	709	10.5	1,076	17.0
Secondary	2,311	36.5	2,467	36.5	2,008	31.6
Post-secondary	1,288	20.3	3,436	50.9	3,051	48.1
No response	1	0.0	144	2.1	209	3.3
***Have a debilitating chronic disease***						
Yes	1,949	30.8	1,732	25.6	2,132	33.6
No	4,382	69.2	5,024	74.4	4,212	66.4
***Income (baht per month)***						
No Income	1,012	16.0	n.a	n.a	474	7.5
< 3,500	167	2.6	n.a	n.a	704	11.1
3,500—10,000	1,021	16.1	n.a	n.a	1,575	24.8
10,001 – 15,000	724	11.5	n.a	n.a	1,089	17.2
15,001 – 30,000	642	10.1	n.a	n.a	1,177	18.5
30,001 – 50,000	188	3.0	n.a	n.a	452	7.1
> 50,000	118	1.9	n.a	n.a	436	6.9
No response	2,459	38.8	n.a	n.a	437	6.9
***Exposed to a PA campaign***						
Yes	1,966	31.1	1,819	26.9	2,997	47.2
No	4,365	68.9	4,937	73.1	3,347	52.8
***Joined the PA campaign***						
Yes	462	7.3	n.a	n.a	759	12.0
No	5,869	92.7	n.a	n.a	5,585	88.0
***Have access to PA facilities***						
Yes	2,909	45.9	n.a	n.a	4,723	74.4
No	3,422	54.1	n.a	n.a	1,621	25.6

Notes: Formal sector employees include (1) civil servants, (2) politicians, (3) officers, (4) factory workers, and (5) retired civil servants. Informal sector employees include (1) freelancers, (2) professional athletes. Sufficient MVPA: an accumulation of 75 min of vigorous PA per week or a 150-min combination of vigorous and moderate PA per week. Abbreviations: *MVPA* Moderate-to-vigorous physical activity, *SPA* Surveillance on physical activity

### Inequality in the proportion of the population with sufficient MVPA

The first indicator of PA inequality in this study is the proportion of population with sufficient MVPA. PA inequality was measured by the proportion of the sample population meeting the WHO guidelines, classified by various characteristics. This type of analysis is similar to most previous studies on PA inequality that focused on PA disparities among different groups of the population. We also calculated the relative difference in the proportion of MVPA sufficiency by sub-population to identify which groups are most vulnerable.

The findings of this study show that the Covid-19 containment measures in Thailand during 2020–21 widened PA inequality, as shown by declining proportion meeting the recommended WHO guidelines in 2020 and 2021. Before the pandemic, the proportion of Thai adults with sufficient MVPA was approaching the national target of 80%. With the strict confinement orders during the first waves of the Covid-19 epidemic, the overall proportion of Thai adults who met the recommended guidelines dropped from 74.3% in 2019 to 56.7% in 2020. As health promotion campaigns were intensified in 2020 (e.g., “*Fit From Home*”), the proportion with sufficient MVPA slightly increased, to 65.5% in 2021 (Table 2). The Fit from Home campaign itself aimed at providing physical activity related information and guidelines to Thai population, particularly on how to stay active during the containment periods. The proportion of adults meeting the recommended guidelines before Covid-19 was lowest among the following: (1) No income (63.2%); (2) The unemployed (67.4%) and (3) Those who resided in Northeast region (68.7%). During the first wave of Covid spread in Thailand (early 2020), those who were unemployed (51.6%) were the most vulnerable (to MVPA insufficiency), followed by individuals residing in Bangkok (52.1%) and females (52.5%). In 2021, the proportion of adults meeting the recommended PA guidelines was the lowest among individuals with no income (55.3%), unemployed (57.4%), and the elderly (58.3%) (Table 2).

**Table 2:** Inequality in the proportion of population with sufficient MVPA: 2019–2021

Characteristics	SPA2019 (*n* = 6,331)						SPA2020 (*n* = 6,756)						SPA2021 (*n* = 6,344)					
	%	S.D	95% CI		Diff.	Chi- square	%	S.D	95% CI		Diff.	Chi- square	%	S.D	95% CI		Diff.	Chi- square
			Lower	Upper					Lower	Upper					Lower	Upper		
***Sex***																		
Male	76.1	42.7	74.6	77.6	3.6	26.905 ***	60.7	48.8	59.1	62.4	8.2	44.665 ***	68.6	46.1	67.0	70.3	5.9	32.703 ***
Female	72.5	44.7	71.0	74.1			52.5	49.9	50.8	54.2			62.7	48.3	61.0	64.3		
***Age group (years)***																		
Yong adult (18–24)	69.2	46.2	65.5	72.8	6.1	11.409 **	56.4	49.6	53.8	59.0	3.7	2.187	63.4	48.2	60.4	66.4	9.7	40.105 ***
Prime working age (25–59)	75.3	43.1	74.0	76.6			57.0	49.5	55.6	58.4			68.0	46.5	66.6	69.4		
Older adults (60 +)	73.4	44.2	71.1	75.6			53.3	49.9	48.5	58.1			58.3	49.0	55.5	61.2		
***Region***																		
North	71.1	45.3	68.7	73.5	11.8	83.640 ***	57.3	49.5	55.1	59.5	8.9	21.226 ***	65.9	47.0	63.7	68.2	8.6	14.038 **
Central	80.5	39.6	78.5	82.5			53.9	49.8	51.8	56.1			64.9	47.7	62.8	67.0		
Northeast	68.7	46.4	66.4	71.0			61.0	48.8	58.0	64.0			67.4	46.9	64.2	70.6		
South	78.7	41.0	76.5	81.0			60.4	48.8	57.3	63.5			69.5	45.9	66.3	72.6		
Bangkok	70.0	45.9	66.2	74.0			52.1	50.0	48.7	55.6			60.9	48.7	57.7	65.1		
***Area of residence***																		
Urban	74.2	43.8	72.7	75.6	0.1	0.067	55.4	49.7	53.9	56.9	3.7	7.364 **	64.7	47.7	63.2	66.1	2.5	4.311 *
Rural	74.3	43.7	72.7	75.9			59.1	49.2	57.1	61.1			67.2	46.7	65.2	69.2		
***Occupation***																		
Agriculture	83.1	37.5	80.9	85.4	15.7	89.552 ***	68.9	46.4	64.6	73.2	17.3	52.967 ***	72.6	44.1	70.0	76.3	15.2	67.187 ***
Formal sector	73.3	44.3	71.0	75.9			56.7	49.5	54.8	58.6			66.3	47.3	64.3	68.2		
Informal sector	75.1	43.2	73.5	76.8			56.4	49.6	54.5	58.2			67.6	46.8	65.6	69.5		
Unemployed	67.4	46.9	65.1	69.8			51.6	50.0	48.1	55.2			57.4	49.3	54.5	60.3		
***Education***																		
Primary or less	73.9	44.0	72.2	75.5	1.2	0.930	53.7	49.9	50.1	57.4	3.9	39.340 ***	59.5	49.1	56.5	62.4	8.0	95.627 ***
Secondary	74.3	43.8	72.5	76.0			57.6	49.4	55.6	59.6			67.3	46.9	65.3	69.4		
Post-secondary	75.1	43.3	72.7	77.4			56.8	49.5	55.1	58.4			67.5	46.9	65.8	69.1		
***Have a chronic disease***																		
Yes	75.8	42.9	73.9	77.7	2.2	2.471	62.0	48.5	59.7	64.3	7.2	38.613 ***	64.5	47.7	62.5	66.6	1.5	0.806
No	73.6	44.1	72.2	74.9			54.8	49.7	53.4	56.2			66.0	47.2	64.6	67.5		
***Income (baht per month)***																		
No Income	63.2	48.2	60.3	66.2	15.0	21.530 ***	n.a	n.a	n.a	n.a	n.a	n.a	55.3	49.8	50.8	59.8	16.6	156.721 ***
< 3,500	71.3	45.4	64.3	78.2			n.a	n.a	n.a	n.a			60.7	48.9	57.0	64.3		
3,500—10,000	73.0	44.4	70.2	75.7			n.a	n.a	n.a	n.a			68.5	46.5	66.2	70.8		
10,001 – 15,000	71.5	45.2	68.3	74.8			n.a	n.a	n.a	n.a			65.0	47.7	62.2	67.9		
15,001 – 30,000	75.9	42.8	72.5	79.2			n.a	n.a	n.a	n.a			69.2	46.2	66.5	71.8		
30,001 – 50,000	78.2	41.4	72.2	84.1			n.a	n.a	n.a	n.a			71.9	45.0	67.7	76.1		
> 50,000	77.1	42.2	69.4	84.8			n.a	n.a	n.a	n.a			71.1	45.4	66.8	75.4		
***Exposed to a PA campaign***																		
Yes	78.7	40.9	76.9	80.5	6.5	30.001 ***	62.7	48.4	60.5	65.0	8.3	37.300 ***	70.3	45.7	68.7	71.9	9.1	57.389 ***
No	72.2	44.8	70.9	73.6			54.4	49.8	53.0	55.8			61.2	48.7	59.6	62.9		
***Joined the PA campaign***																		
Yes	84.8	35.9	81.6	88.1	11.4	29.262 ***	n.a	n.a	n.a	n.a	n.a	n.a	70.9	45.5	67.6	74.1	6.1	10.950 ***
No	73.4	44.2	72.3	74.6			n.a	n.a	n.a	n.a			64.8	47.8	63.5	66.1		
***Have access to PA facilities***																		
Yes	72.3	44.7	70.7	74.0	-3.6	10.447 ***	n.a	n.a	n.a	n.a	n.a	n.a	67.8	46.7	66.4	69.1	8.8	41.360 ***
No	75.9	42.8	74.5	77.3			n.a	n.a	n.a	n.a			59.0	49.2	56.6	61.4		

Notes: No response was excluded from the analysis. Diff: between-group relative difference. Formal sector employees include (1) civil servants, (2) politicians, (3) officers, (4) factory workers, and (5) retired civil servants. Informal sector employees include (1) freelancers, (2) professional athletes. Sufficient MVPA: an accumulation of 75 min of vigorous PA per week or a 150-min combination of vigorous and moderate PA per week. Abbreviations: *MVPA* Moderate-to-vigorous physical activity, *SPA* Surveillance on physical activity. ***Significant at *p*-value < 0.001, **Significant at *p*-value < 0.01, *Significant at *p*-value < 0.05

Although inequality existed in all sub-populations, the groups with the lowest proportion meeting the WHO guidelines were the unemployed and those with no income. PA inequality was highest among those two sub-populations, as shown by the largest relative difference between the groups, both in the pre-Covid and epidemic periods. The gap in the proportion accumulating sufficient MVPA was the largest between those employed in the agricultural sector and the jobless (15.7, 17.3 and 15.2% in 2019–2021, respectively). The gap in MVPA sufficiency was also the largest among individuals from different income levels, particularly between those who earned the most and those with no income (15 and 16.6% in 2019 and 2021, respectively) (Table 2).

### Inequality in the average cumulative minutes of MVPA

The second indicator used to determine inequality in this study was the average cumulative minutes of MVPA per week. Similar to the previous indicator, PA inequality was defined as the disparity in minutes of MVPA among different groups of the population. We identified the most vulnerable groups by selecting the largest difference in the cumulative minutes of MVPA between groups. We also observed how the differences in the cumulative minutes changed between pre-Covid and epidemic periods.

The findings of the study showed that the Covid-19 epidemic and response in Thailand has affected the level of PA of the population, not only in terms of the proportion of population who can meet the recommended PA guidelines, but also in the cumulative minutes of MVPA collected weekly. The overall PA of Thai adults dropped significantly, from 559 min in 2019 to 506 min in 2020, and continued to decline to 496 min in 2021 (Table 3). Before Covid-19 emerged, the sub-populations with the lowest MVPA were the following: (1) No income (315 min); (2) Unemployed (351 min); and (3) Earning < 3,500 baht per month (387 min). As the SPA did not collect income data in 2020, unemployed individuals took first place among the least active (423 min), followed by females (432 min), older adults (433 min), and those who resided in Bangkok (434 min). In 2021, the three sub-populations with the lowest cumulative minutes of MVPA were those with no income (382 min), unemployed (418 min) and Bangkok residents (424 min) (Table 3).

**Table 3:** Inequality in the average cumulative minutes of MVPA of the Thai population: 2019–2021

Characteristics	**SPA2019**						**SPA2020**						**SPA2021**						Relative diff (Gini)	
	(*n* = 6,331)						(*n* = 6,756)						(*n* = 6,344)							
	Mean	S.D	95% CI		Mean diff. test	Gini	Mean	S.D	95% CI		Mean diff. test	Gini	Mean	S.D	95% CI		Mean diff. test	Gini	2019–2020	2019–2021
			Lower	Upper					Lower	Upper					Lower	Upper				
***Overall MVPA (min/week)***	559	682	542	576		0.416	506	654	491	522		0.440	496	610	481	511		0.487	5.8	17.1
***Sex***																				
Male	614	589	589	639	6.265 ***	0.453	583	695	560	607	9.795 ***	0.466	531	614	509	554	3.576 ***	0.490	3.0	8.3
Female	507	656	480	529		0.440	432	597	411	452		0.448	472	605	451	493		0.448	1.8	1.8
***Age group (years)***																				
Yong adult (18–24)	486	616	438	535	22.289 ***	0.522	535	691	498	573	5.173 ***	0.504	443	595	404	481	11.715 ***	0.520	-3.5	-0.3
Prime working age (25–59)	599	705	578	620		0.433	507	647	489	525		0.452	524	618	505	543		0.480	4.4	10.7
Older adults (60 +)	475	628	443	507		0.390	433	590	375	491		0.549	461	589	425	496		0.531	40.7	36.2
***Region***																				
North	431	595	399	463	66.648 ***	0.476	504	652	474	534	5.718 ***	0.471	535	660	503	567	4.983 **	0.511	-0.9	7.4
Central	727	784	687	767		0.466	497	664	468	526		0.478	500	621	472	527		0.505	2.4	8.3
Northeast	453	590	424	482		0.464	572	676	530	614		0.465	512	589	471	553		0.512	0.1	10.3
South	688	743	649	728		0.471	533	654	491	574		0.473	502	551	464	541		0.482	0.4	2.4
Bangkok	398	485	357	439		0.492	434	595	393	476		0.514	424	548	388	461		0.514	4.5	4.3
***Area of residence***																				
Urban	503	618	482	524	-7.017 ***	0.422	490	645	471	509	-2.294 ***	0.453	476	596	458	495	-3.247 **	0.489	7.3	15.9
Rural	625	745	598	652		0.460	543	666	515	571		0.467	546	633	519	574		0.496	1.5	7.9
***Occupation***																				
Agriculture	814	789	766	862	83.114 ***	0.452	698	717	631	766	17.519 ***	0.463	617	668	561	672	17.018 ***	0.495	2.5	9.7
Formal sector	484	606	449	520		0.387	474	626	450	498		0.460	461	544	439	484		0.486	18.8	25.7
Informal sector	611	749	582	639		0.461	534	674	508	559		0.458	553	683	524	582		0.502	-0.6	8.8
Unemployed	351	410	330	371		0.441	423	602	381	466		0.522	418	534	386	450		0.534	18.3	21.1
***Education***																				
Primary or lower	576	712	549	603	8.517	0.460	523	694	472	575	8.872	0.530	492	682	451	533	5.256	0.537	15.2	16.8
Secondary	585	698	557	614	***	0.464	560	691	533	588	***	0.458	531	649	502	559	**	0.504	-1.2	8.5
Higher	473	572	442	505		0.445	468	612	447	488		0.452	484	554	464	503		0.470	1.5	5.6
***Have a debilitating*** ***chronic disease***																				
Yes	520	649	491	549	-3.078	0.457	569	675	536	601	4.463	0.461	509	646	481	537	0.945	0.510	0.8	11.5
No	576	695	555	596	**	0.435	487	644	496	505	***	0.455	496	591	478	514		0.484	4.5	11.1
***Income (baht per month)***																				
No Income	315	390	291	339	32.448 ***	0.370	n.a	n.a	n.a	n.a	n.a	n.a	382	530	334	430	7.685 ***	0.551	n.a	48.6
< 3,500	387	419	323	451		0.488	n.a	n.a	n.a	n.a		n.a	476	666	427	526		0.548	n.a	12.4
3,500—10,000	532	670	491	573		0.495	n.a	n.a	n.a	n.a		n.a	544	647	513	576		0.497	n.a	0.5
10,001 – 15,000	562	704	511	613		0.517	n.a	n.a	n.a	n.a		n.a	531	653	492	570		0.515	n.a	-0.3
15,001 – 30,000	562	688	508	615		0.506	n.a	n.a	n.a	n.a		n.a	486	582	453	520		0.495	n.a	-2.3
30,001 – 50,000	562	652	468	655		0.513	n.a	n.a	n.a	n.a		n.a	541	544	491	592		0.474	n.a	-7.8
> 50,000	550	821	400	700		0.565	n.a	n.a	n.a	n.a		n.a	497	495	451	544		0.470	n.a	-16.9
***Exposed to PA campaign***																				
Yes	556	637	528	584	-0.181	0.443	550	647	521	580	3.380 **	0.444	542	628	519	564	5.642 ***	0.475	0.1	7.1
No	560	701	539	580		0.440	490	655	471	508		0.456	455	590	435	475		0.508	3.7	15.4
***Joined the PA campaign***																				
Yes	583	627	525	640	0.847	0.439	n.a	n.a	n.a	n.a	n.a	n.a	538	604	495	581	2.034	0.487	n.a	10.9
No	557	686	539	574		0.425	n.a	n.a	n.a	n.a		n.a	490	611	474	506		0.491	n.a	15.5
***Have access to PA facilities***																				
Yes	482	612	460	504	-8.387	0.439	n.a	n.a	n.a	n.a	n.a	n.a	509	615	491	526	2.813 **	0.475	n.a	8.2
No	624	730	599	648	***	0.449	n.a	n.a	n.a	n.a		n.a	459	594	430	488		0.534	n.a	18.9

Notes: No response was excluded from the analysis. Formal sector employees include (1) civil servants, (2) politicians, (3) officers, (4) factory workers, and (5) retired civil servants. Informal sector employees include (1) freelancers, (2) professional athletes. Sufficient MVPA: an accumulation of 75 min of vigorous PA per week or a 150-min combination of vigorous and moderate PA per week. Abbreviations: *MVPA* Moderate-to-vigorous physical activity, *SPA* Surveillance on physical activity. Mean difference test was performed by using independent t-test (2 categories variable) and one way ANOVA (> 2 categories variable). *significant at *p*-value 0.05, ** significant at *p*-value 0.01, *** significant at *p*-value 0.001

In the pre-epidemic situation, occupation-based inequality in PA was largest, particularly between those who worked in agriculture versus the unemployed, marked by between-group difference (Δ) of 464 min. Regional and SES disparities in PA also raise a concern, since within-group inequality is also considered high, particularly between those who resided in the Central region versus Bangkok (Δ 329 min), and between the highest income earners versus those with no income (Δ 247 min). During the epidemic, the difference in the cumulative minutes decreased because there was a reduction in the overall PA of the population. However, PA inequality remained the largest in the sub-population who were unemployed (compared to those working in agriculture, Δ 275 and 199 min in 2020 and 2021, respectively), and those with no income (compared with the highest earners, Δ 162 min in 2021). Regional disparities were also relatively high during the epidemic, with Bangkok residents still having the lowest MVPA accumulation throughout the three years of the survey (Δ 138 min in 2020 and 111 min in 2021).

### Applying the Gini coefficient to assess PA inequality

The third indicator employed to measure PA inequality in this study is the Gini coefficient. Gini is a summary statistic of the Lorenz curve [32], which shows the cumulative percentage of the population on the x axis and cumulative share of PA of the population on the y axis. A greater Gini coefficient (i.e., closer to 1) indicates greater inequality in PA distribution. By using the cumulative minutes of MVPA as the base data, this study found that the Gini coefficient of overall MVPA of Thai adults during the three rounds of the SPA tended to increase in a concerning level, from 0.416 in 2019 to 0.440 and 0.487 in 2020 and 2021, respectively.

The effects of Covid-19 on PA inequality can be seen from the Gini coefficient in Table 3, by comparing 2019 (pre-epidemic) to 2020 and 2021. Before the epidemic, the highest level of PA inequality was found among the sub-populations with the highest income (0.565), young adults (0.522), and those earning 10,001 – 15,000 baht per month (0.517). As the SPA did not collect income data in 2020, between-group inequality was largest among the older adults (0.549), those who attained primary education or lower (0.530), and the unemployed (0.522). With the availability of income data in 2021, PA inequality was found to be highest among those with no income (0.551), those earning < 3,500 baht per month (0.548), and those who had attained primary education or less (0.537).

This study determined the most vulnerable groups affected by the Covid-19 epidemic from the largest relative difference of Gini (Table 3). PA inequality was the greatest among individuals with no income, marked by a relative difference of Gini (Δ) of 48.6 in 2021. The group of seniors was also severely affected by the epidemic (Δ 40.7 and 36.2 in 2020 and 2021, respectively) as PA inequality among this group was the lowest (0.39) before Covid-19 (2019), and significantly increased (> 0.5) during the epidemic (2020 and 2021). Those who worked in the formal sector were also among the most vulnerable (Δ 25.7 in 2021) as they lost most of their occupation-related PA during the Covid containment measures.

### Combining the three parameters to determine the most vulnerable groups of the population

While the level of inequality in each sub-population was established (Table 3), it is not clear which groups of the population are the most vulnerable. Different groups were classified as most vulnerable based on the availability of data each year and the indicator being used. Therefore, the determination of vulnerability due to PA inequality in this study involved averaging the ranks from three parameters: Percent sufficient MVPA, minutes of MVPA, and the Gini coefficient. This study identified the most disadvantaged groups of the population as those whose PA was affected the most, based on three conditions: Least MVPA minutes, lowest proportion of sufficient MVPA, and ranked highest in the PA inequality index (Gini). Accordingly, the most vulnerable populations were identified as follows: (1) Those with no income; (2) The unemployed; (3) Those who have no access to PA facilities; (4) older persons age 60 + years; and (5) Those earning < 3,500 baht per month. Further, residents of Bangkok, young adults aged 18–24 years, those who attained primary education or less, those who had no exposure to a PA awareness campaign, and those who had a debilitating chronic disease also had elevated risk of PA deficiency (Table 4).

**Table 4:** Determination of the most vulnerable group of the population

Population characteristics	Rank of % MVPA	Mean ± SD	Rank of minutes MVPA	Mean ± SD	Rank of Gini index	Average rank	
	(min–max)		(min–max)		(max–min)		
***Sex***							
Male	27	+	26	+	22	27	
Female	9	-	9	-	34	16	
***Age group (years)***							
Yong adults (18–24)	10	-	4	-	7	7	*
Prime working age (25–59)	25	+	23	+	28	29	
Older adults (60 +)	3	-	7	-	6	4	**
***Region***							
North	16	+	27	+	11	18	
Central	14	-	18	+	14	11	
Northeast	21	+	22	+	10	17	
South	29	+	19	+	27	27	
Bangkok	7	-	3	-	9	6	*
***Area of residence***							
Urban	12	-	11	-	23	11	
Rural	19	+	32	+	18	23	
***Occupation***							
Agriculture	34	+	34	+	19	32	
Formal sector	18	+	8	-	25	15	
Informal sector	23	+	33	+	16	24	
Unemployed	2	-	2	-	5	2	**
***Education***							
Primary or less	5	-	15	-	3	8	*
Secondary	20	+	24	+	15	19	
Higher	22	+	12	-	32	22	
***Have a debilitating chronic disease***							
Yes	11	-	21	+	12	10	*
No	17	+	16	+	26	19	
***Income (baht per month)***							
No Income	1	-	1	-	1	1	**
< 3,500	6	-	10	-	2	5	**
3,500—10,000	26	+	31	+	17	26	
10,001 – 15,000	15	-	25	+	8	13	
15,001 – 30,000	28	+	13	-	20	21	
30,001 – 50,000	33	+	29	+	31	34	
> 50,000	32	+	17	+	33	30	
***Exposed to PA campaign***							
Yes	30	+	30	+	30	33	
No	8	-	5	-	13	9	*
***Joined the PA awareness campaign***							
Yes	31	+	28	+	24	31	
No	13	-	14	-	21	13	
***Have access to PA facilities***							
Yes	24	+	20	+	29	25	
No	4	-	6	-	4	3	**

Notes: No response was excluded from the analysis. **The five most vulnerable groups of the population. *The ten most vulnerable. ( +)/(-) mean ± SD of a particular group is higher or lower than the mean of the population

**Fig. 1:**
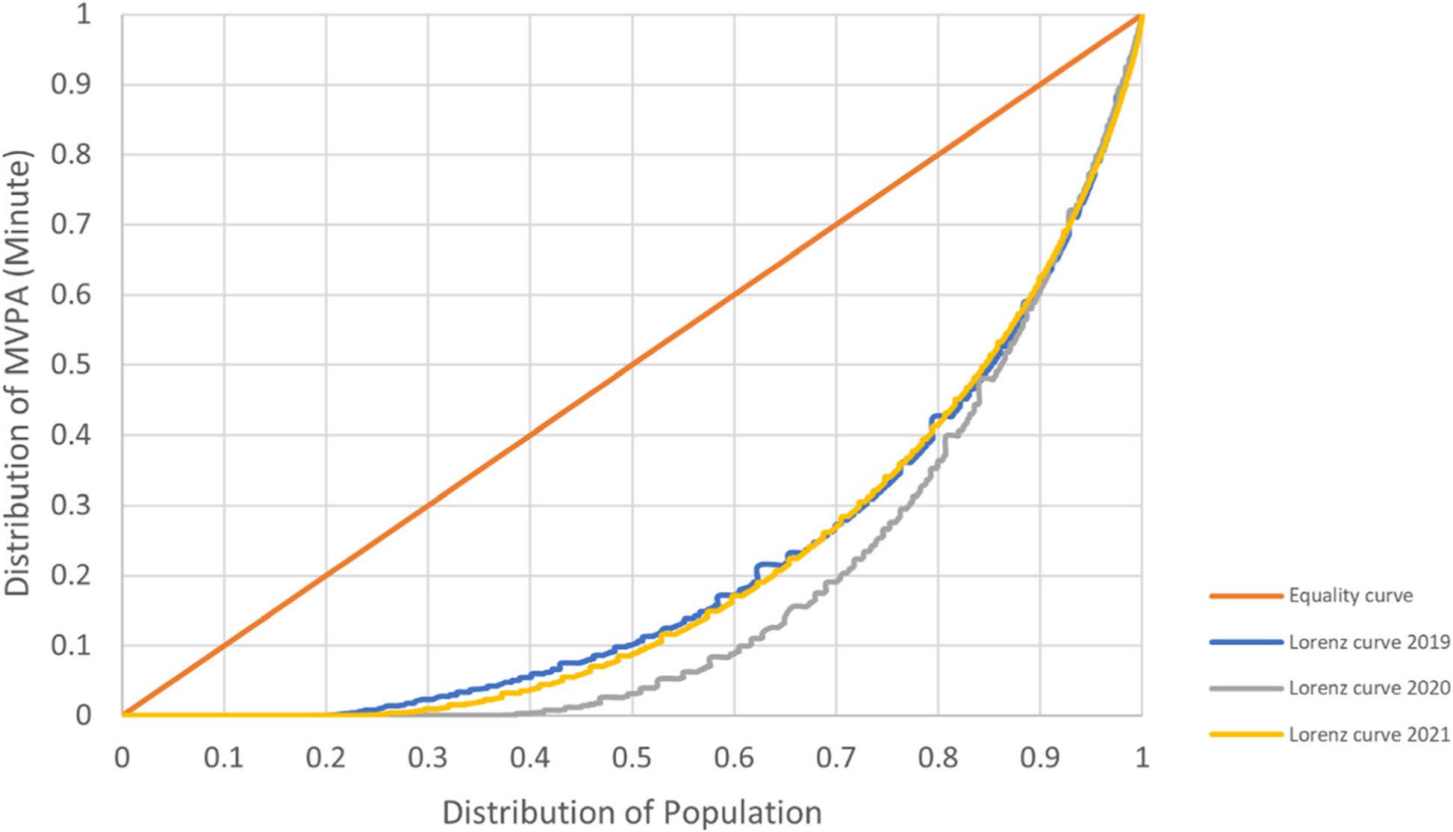
The Lorenz curve of the distribution of minutes of PA by year

While PA inequality was identified before Covid, the pandemic has widened the gap. In Thailand, one piece of evidence for an effect of Covid-19 on PA inequality derives from the wider area under the equality line in the Lorenz curve (Fig. 1). The largest area under the equality line indicates the highest inequality. One of the advantages of using the Lorenz curve to estimate inequality is that it portrays the distribution of population against the distribution of minutes of MVPA, a key point of interest in this study. From the Lorenz curve of 2019, about half the population accounted for only 10% of the cumulative minutes of MVPA – which, in a perfectly equal scenario, 50% of the population would account for 50% of the cumulative minutes of MVPA. The 9^th^ percentile of the 2019 Lorenz curve corresponds to 60% of the cumulative MVPA minutes, which means that the top 10% of the Thai population accumulated 40% of total MVPA time. The Covid-19 epidemic has worsened PA inequality, in that half the population accounted for only 3% of total MVPA in 2020, and 8% in 2021. The 9^th^ percentile of the 2020 Lorenz curve corresponds to 61% in 2020 and 62% in 2021, suggesting that the top 10% of the Thai population accumulated 39% and 38% of the MVPA minutes, respectively.

## Discussion

A lot of evidence has been accumulating which points to a significant decline in PA during the Covid-19 pandemic, particularly during the strict containment measures imposed by national, state/provincial, and municipal governments around the world. The shape of the Lorenz curve corresponds with the prevalence of MVPA, with a higher degree of inequality (shown as the area under the equality line in the 2020 and 2021 Lorenz curves) occurring when the prevalence of sufficient MVPA was lowest during the first wave of Covid-19 epidemic. After Thai adults adjusted to the Covid restrictions, they managed to modify their PA routines, and the proportion of population with sufficient MVPA increased in 2021 [8]. However, the Gini coefficient in 2021 was the highest of the three years. This suggests that, although the proportion of the Thai population who achieved the recommended level of MVPA increased, inequality remained. The findings imply that only a small part of the adult population successfully adjusted their PA (e.g., shifting from outdoor to indoor PA, buying equipment for home-based PA, etc.) and accumulated a higher level of MVPA, whereas the rest were unable to regain their pre-epidemic level of PA. The gap in the distribution of the population who achieved the highest and lowest level of PA was largest in 2021, as indicated by 16.9 points of relative difference of the Gini coefficient from the base (2019), compared to 2020, with only 5.7 points (Table 3).

Previous studies have consistently reported that socio-economic inequality in PA was driven by income, education, and occupation differentials. Higher-SES individuals are more likely to have more opportunity to engage in recreational PA, whereas their lower-SES counterparts engaged mostly in work-related PA [16, 18]. Among the higher-SES, inequality was mostly related to access to leisure PA, as increased income is generally correlated with increased work hours and reduced leisure time [4]. In this study, however, the most vulnerable group was identified as those with no income and unemployed, shown by the lowest rank (1 and 2) in the Table 4. In addition, lower-SES individuals (earning income < 3,500 baht per month, attained primary education or less) were also ranked the 5^th^ and 8^th^ most vulnerable groups, i.e., where PA inequality was highest. In Thailand, the Covid-19 restrictions had the most impact on the lower-SES portion of the population, as many were laid off or had reduced work hours. Thus, inequality in MVPA widened by the limited work-related PA for that group. Moreover, recreational PA was probably not a lifestyle priority for this group since they had to conserve time and financial savings to cover essential living expenses. The Covid-19 epidemic has also disproportionately affected the lower-income population due to less access to prevention technology or urgent medical care when ill [41]. Even those individuals who were able to remain gainfully employed during the epidemic sustained a drop in income, but many had savings to fall back on. That said, surely the unemployed with little savings sustained the greatest hardship during Covid-19 lock-downs and other containment measures [42, 43].

The third most vulnerable groups of the population (i.e., where PA inequality is the highest) are those who have no access to PA facilities. Individuals who had no exposure to the various PA awareness campaigns were also among the more vulnerable groups (rank 9). These results are consistent with previous studies, that PA participation is determined by access to PA facilities and health-related information [3, 17]. Further, half of sample (52.8%) had never been exposed to any PA awareness campaigns. The findings of this study point to the importance of improving access to PA-related information and facilities in order to increase the PA level of the most vulnerable group, as it aligned with the WHO “*Fair Play*” initiative [13].

Older (60 + years) and younger (18–24 years) persons are also among the ten most vulnerable groups of the population (rank 4 and 7, respectively). For older persons, this finding is to be expected since the elderly are among the most severely-affected populations by Covid-19 generally. While PA inequality was relatively low before the epidemic (0.39 in 2019), the distribution of MVPA among the seniors showed a great discrepancy during the epidemic. Many older persons lost most of their PA opportunities because they relied on public amenities and collective PA (e.g., public parks, group exercise) which were closed as one of the many Covid containment measures [44, 45]. Older persons also had the highest mortality among Covid-19 patients in many parts of the world [46, 47] and, thus, were strongly recommended to limit outdoor activities during outbreaks. In this study, PA inequality among the elderly was the highest in 2020 because only a small portion of seniors were able to localize their PA by using facilities at home, or obtaining family support for PA as a substitute for their peer group.

Before the epidemic, PA inequality among young adults (Gini coefficient 0.522) was likely to be influenced by an increasing demand for work- and school-related activities, and correspondingly fewer PA opportunities [48]. However, with the advent of Covid-19 and the containment measures throughout the country, the gap between the most- and least-active young adults slightly narrowed (Gini coefficient 0.504). The degree of inequality increased in 2021 (Gini coefficient 0.520) because some young adults successfully adjusted their PA routine, whereas the rest remained sidelined by the epidemic. Disadvantaged young adults were also constrained by lack of access to PA facilities which may also be related to SES differentials.

Urban–rural and regional disparities in PA have been documented worldwide. With regard to exposure to health promotion initiatives and access to PA amenities, urban dwellers and those who reside in the more developed regions of the world accumulated more PA minutes than their rural counterparts [1, 6, 16–18]. However, this study found PA inequality in all regions of Thailand, with the largest gaps in Bangkok. Residents of Bangkok were among the most vulnerable populations (rank 6) during the epidemic given the high spread of infection in the densely-populated parts of the city. People in megacities such as Bangkok also have higher rates of contact and proximity with strangers through mass transit and related transport systems [49]. The government tried to prevent outbreaks by ordering the closure of a wide range of establishments, and mandating social distancing on mass transit and any other place where people tend to form crowds. Businesses and government agencies were encouraged to implement “*work-from-home*” policies wherever possible. Given the relatively lower priority for PA, it is not surprising that PA access and opportunities of residents of Bangkok were disproportionately limited during Covid-related containment measures.

Approximately 25–33% of the sample in this study suffered from chronic diseases (i.e., hypertension, diabetes, obesity, arthritis, and cardiovascular diseases) at different stages of illness. Although PA was often prescribed to patients with chronic diseases, however, the presence of debilitating condition determines PA inequality among this group (rank 10). The existing literatures documented, barriers of PA participation among patients with chronic disease were driven by mobility difficulties, severe pain, hearing problems, visual impairment, and/or multimorbidity [50–53].

Previous studies have consistently reported that gender inequality in PA occurs because the nature of sports and exercise favors males [16, 18]. Females appeared to be at a natural disadvantage because the proportion who could comply with the recommended MVPA was consistently lower than their male counterparts, resulting in fewer minutes of MVPA [1, 5, 38]. WHO, through its “*Fair Play*” campaign [13] also emphasized the need to address inequity in access and opportunity for PA, particularly among women and other groups with relatively lower MVPA. Interestingly, this study found that gender was not significantly associated with vulnerability during the epidemic (i.e., males ranked 27 and females ranked 16). It is true that PA inequality exists, but it was not a factor behind the large discrepancy within and between sexes. Compared to females, a higher degree of inequality in minutes of MVPA was observed among males, and inequality slightly worsened during the epidemic. The finding suggests that men were more severely affected by the epidemic restrictions when they lost the access to gyms, team sports, and organized outdoor PA; only a minority were able to successfully adjust to the “*new normal*.” Among females, the gap in the MVPA minutes was lower, because female PA – either in terms of access or participation – was quite uniform already. Both during the epidemic and in the pre-Covid era, female adults were mostly constrained by their culturally-prescribed role as ‘homemaker,’ plus a reluctance to venture out in the evening to engage in PA [54]. Indeed, when examining the three parameters (percent sufficient MVPA, MVPA minutes, and Gini coefficient) by gender, females were clearly more vulnerable than the males (Table 4).

This study has contributed to the analysis of PA inequality in several ways. First, given that the data are from nationally-representative samples, the results of this study should reflect the actual situation in the country. Utilizing a time series of data adds to the strength of results, particularly in generating evidence of the effect of the Covid-19 containment measures by comparing pre-Covid and epidemic inequality. Secondly, the use of the Gini coefficient and Lorenz curve to describe PA inequality provides substantial evidence of the level of inequality, and also shows which sub-groups of the population are most vulnerable. Thirdly, unlike previous studies which mostly relied on a single indicator, the determination of vulnerability in this study is based on three parameters (proportion having sufficient MVPA, cumulative MVPA minutes, and Gini coefficient). However, PA inequalities was also examined in each indicator, to show that the vulnerable population resulted from the single-indicator analysis was varied, depends on the data availability and population distribution. Fourth, this study demonstrated a simple yet strong methodology for analyzing PA inequality that can be applied in many settings, since the determination of inequality involved commonly-available data (i.e., proportion of sufficient MVPA and cumulative MVPA minutes).

Nevertheless, several limitations of this study should also be acknowledged. First, this study recorded zero minutes of MVPA (as shown in the Lorenz curve) because the GPAQ measures PA only when at least ten minutes is accumulated for an activity session. Although the cut-off point in defining a sufficient level of MVPA used the new 2020 WHO guidelines on PA, there was no update for the GPAQ at the time of this report. Secondly, this study did not include children and youth in the analysis, although it is widely known that this group are at increased risk of low PA. Third, considering the sensitivity of revealing personal income level (i.e., there were many missing values for this variable), the findings probably do not accurately reflect the actual contribution of income in generating MVPA inequality. Further, some respondents may have underreported actual income to avoid taxation. However, this study also measured MVPA inequality by educational attainment and occupation, which can be considered proxies for income, and found that the disparity for MVPA was higher among those at lower SES levels.

## Conclusions

The Covid-19 epidemic has disproportionately affected sub-groups of the Thai population and worsened MVPA inequality. In all sub-populations – classified by age, sex, SES, access to information and PA facilities –inequality is at concerning level over time. By using a combination of three parameters, the most vulnerable groups were identified as follows: (1) Those with no income; (2) The unemployed; (3) Those who have no access PA facilities; (4) Older persons age 60 + years; and (5) Those earning < 3,500 baht per month. Further, residents of Bangkok, young adults aged 18–24, individuals who attained primary level of education or less, those who had no exposure to a PA awareness campaign, and those who had a debilitating chronic disease were also among the most vulnerable populations. The results of this study indicate that, with the socio-economic turmoil during the epidemic, the vulnerable populations require more assistance than previously. Therefore, future programs and policies should strive to improve PA opportunity for the most affected populations in order to reduce PA inequality.

## Data Availability

The datasets generated and/or analyzed during the current study are available in the TPAK repository: https://tpak.or.th/th/article/522
